# Case Report: Successful Treatment of Acute Generalized Exanthematous Pustulosis With Secukinumab

**DOI:** 10.3389/fmed.2021.758354

**Published:** 2021-12-16

**Authors:** Liangliang Zhang, Qiuyun Xu, Tingting Lin, Shifan Ruan, Mengting Lin, Chengbei Bao, Jing Zhang, Tao Liu, Ting Gong, Chao Ji

**Affiliations:** ^1^Department of Dermatology, The First Hospital of Fujian Medical University, Fuzhou, China; ^2^Department of Medical Affairs, Beijing Novartis Pharma Company Limited, Shanghai, China

**Keywords:** AGEP, secukinumab, biologic, interleukin-17A inhibition, drug eruption

## Abstract

Acute generalized exanthematous pustulosis is a severe, usually drug-related reaction, characterized by an acute onset of mainly small non-follicular pustules on an erythematous base. Most cases of acute generalized exanthematous pustulosis (AGEP) clear quickly with a systemic corticosteroid, but severe or recalcitrant cases may need other systemic therapies. In this case, a man in his 40 s with a history of psoriasis consulted a physician about widespread erythema, pustules, target lesions, and fever after the administration of a quadruple antituberculosis drug. Routine laboratory testing revealed elevated white blood cell count and C-reactive protein. The histopathology showed subcorneal pustules, spongiosis as well as lymphocyte and eosinophils infiltration in the dermis. The patient was diagnosed with definitive AGEP according to the diagnostic score from the EuroSCAR study. Cutaneous lesions especially pustules and erythema multiforme-like lesions on the upper arms and palms are crucial for distinguishing AGEP from Generalized pustular psoriasis. The patient was treated with secukinumab as a result of his failure to respond to topical corticosteroids and constrain of systemic steroids. Remission with secukinumab therapy was safe without increased risks of infections. This case indicates that secukinumab is a potential therapy that can rapidly improve the clinical symptoms of AGEP.

## Introduction

Acute generalized exanthematous pustulosis is a rare disease, therefore there are no high-quality evidence-based therapeutic recommendations. As it is, treatment is mainly potent topical or systemic steroids. However, a minority of cases fail to respond to them, highlighting the need for additional treatment options for these refractory cases (1). Secukinumab is an interleukin (IL)-17 inhibitor that is approved to treat psoriasis. However, the efficacy of secukinumab on acute generalized exanthematous pustulosis (AGEP) is unclear. In this review, we reported the successful use of secukinumab in a patient with AGEP.

## Report of a Case

**Figure 1:**
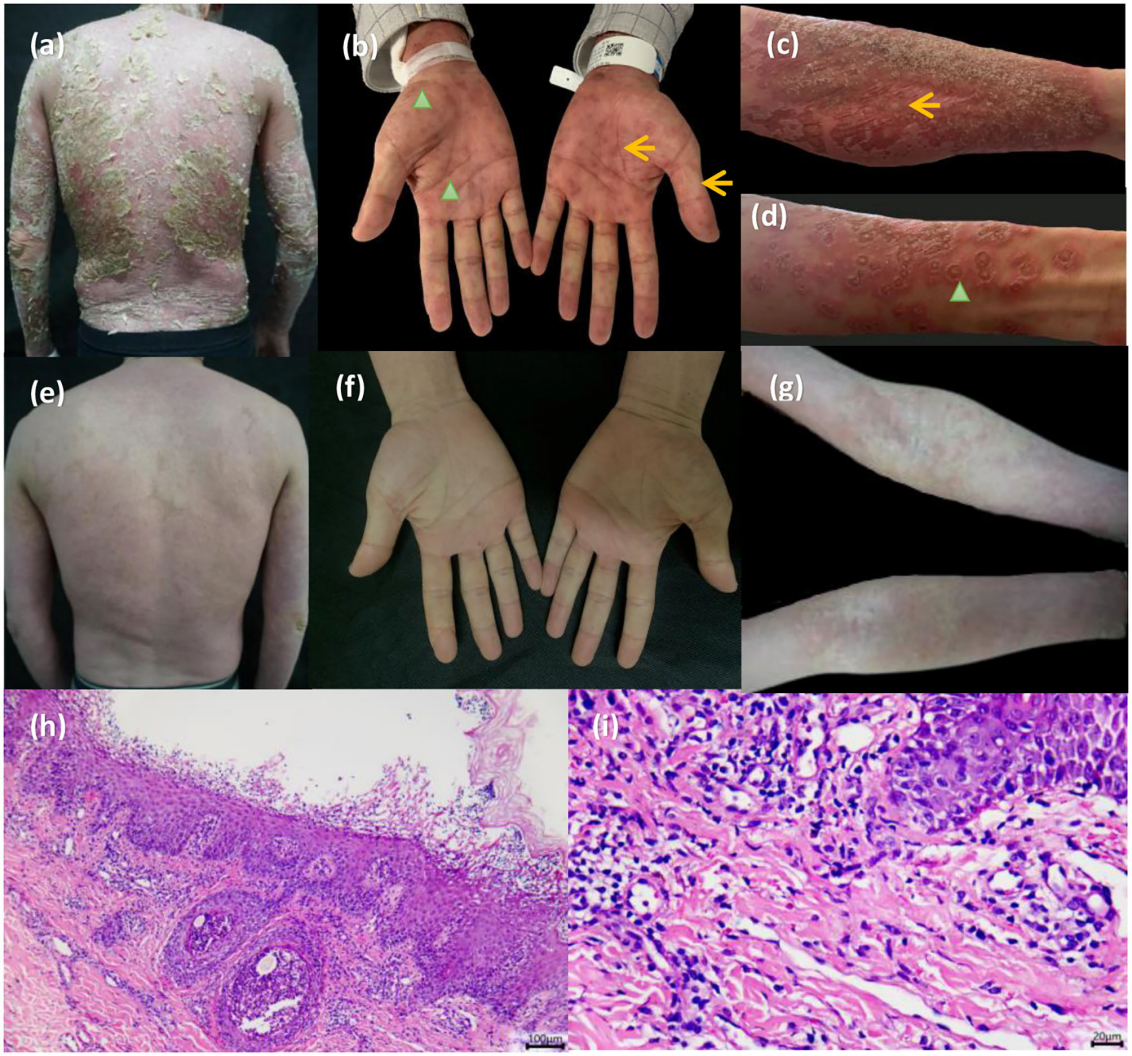
**(a)** The patient presented with diffuse erythema and dried-up lakes of pus on the back. **(b–d)** Pinpoint pustules and erythema multiforme-like lesions were diffusely spread over the upper arms and palms (pinpoint pustules, yellow arrowhead; target lesions, green arrowhead). **(h,i)** Biopsy results demonstrated subcorneal pustules, spongiosis as well as lymphocyte and eosinophils infiltration in the dermis. **(e–g)** All pustules faded 3 days later and erythema substantially darkened.

A 41-year-old man presented with a 1-day history of rapidly growing erythema and pustules on the face, trunk, and extremities. One day before, he took isoniazid, rifampicin, ethambutol, and pyrazinamide to treat pulmonary tuberculosis in lazaretto. At admission, the patient was febrile to a temperature of 40°C. He also had a 2-year history of plaque psoriasis with no history of smoking. Routine laboratory testing revealed white blood cell count of 13,890/μl (normal range, 1,214–5,110/μl) and serum levels of C-reactive protein (CRP) of 7.75 mg/dl (normal range, 0–0.2 mg/dl). Physical examination revealed widespread erythema and dried-up lakes of pus, taking up body surface area of 80%. Pustules and erythema multiforme-like lesions were diffusely spread over the upper arms and palms (Figures 1a–d). Biopsies of pustule and erythema multiforme-like lesions on the arms were performed. The result demonstrated subcorneal pustules, spongiosis as well as lymphocyte and eosinophils infiltration in the dermis (Figures 1h,i).

The patient was assessed using a diagnostic score according to the EuroSCAR study. The final score was nine, which was defined as the definitive AGEP. The patient was subsequently treated with expectant treatment and topic potent steroids for 4 days, followed by no improvement. However, given the patient's femoral head necrosis, systemic glucocorticoid was not recommended. Repeated computed tomography on the chest showed multiple calcifications and thus excluded the possibility of active tuberculosis. Other infections including hepatitis B and hepatitis C were also excluded. The patient received secukinumab 300 mg after being informed of impersonal medical risks (Supplementary Figure). After the first therapy of secukinumab, all pustules faded at day 3, and erythema substantially darkened with defervescence (Figures 1e–g). The Dermatology life quality index (DLQI) decreased rapidly from 25 to 1 at week 4. There were no clinical symptoms and radiological data related to the progression of latent tuberculosis infection.

## Discussion

In this case, the patient had a medication history of quadruple anti-tuberculosis drugs, specific exanthem of erythema multiforme-like lesion, abnormal laboratory results of white blood cell count and C-reactive protein, dermatologic evidence of spongiosis, and subcorneal pustule and eosinophils infiltration. He was then diagnosed with definitive AGEP according to the diagnostic score from the EuroSCAR study. Generalized pustular psoriasis, either the “Annular variant” or “Exanthematic variant,” can also present a similar acute eruption following initiation of medications. The common disease-causing medications include TNF-α inhibitors, lithium, interferons, antimalarials, beta-blockers, rapid withdrawal of systemic corticosteroids. This case provides some clinical reasonings of differential diagnosis between AGEP and GPP. First, cutaneous lesions especially pustules and erythema multiforme-like lesions on the upper arms and palms are crucial for differential diagnosis in this case. Besides, dermatologic evidence of spongiosis, as well as eosinophils infiltration in pathology, favors a diagnosis of AGEP. Finally, the rapid resolution of pustular eruption with secukinumab is suggestive of the diagnosis being AGEP.

Owing to his failure to respond to topical corticosteroids and constrain of systemic steroids, the patient was treated with secukinumab for 1 month. Disease progression was then controlled promptly, with a resolution of pustules and erythema. The great improvement of dermatology-specific health-related quality of life was also observed. Prior to clinical admission to our department, the patient was misdiagnosed as active TB and was sequentially prescribed isoniazid, rifampicin, ethambutol, and pyrazinamide in lazaretto. At admission, active tuberculosis was excluded based on clinical symptoms and results of repeated chest computed tomography. We confirmed that the patient only had latent tuberculosis infection. Thus, this patient did not receive any treatment for his latent TB. The chest radiography was done 1 month later and then every 6 months over a 2-year follow-up. The results showed no change in the lung pathology (calcification). As reported in the literature, patients with latent TB who received no chemoprophylaxis could be safely treated with secukinumab (1, 2).

The underlying mechanism of action of secukinumab on AGEP is related to IL-17. AGEP has been classified as a T cell-related sterile neutrophilic inflammatory response with a predominant T-helper (Th) 1 cell and Th17 (3–5). Th17 cells release IL-17, which affects neutrophil recruitment (6, 7). Therefore, the IL-17 pathway is a potential therapeutic target shown to be implicated in AGEP. Further clinical trials are needed to corroborate the safety and efficacy of secukinumab in AGEP.

## Data Availability Statement

The original contributions presented in the study are included in the article/Supplementary Material, further inquiries can be directed to the corresponding author/s.

## Ethics Statement

The studies involving human participants were reviewed and approved by Ethics Committee of the First Affiliated Hospital of Fujian Medical University. The patients/participants provided their written informed consent to participate in this study. Written informed consent was obtained from the individual(s), and minor(s)' legal guardian/next of kin, for the publication of any potentially identifiable images or data included in this article.

## Author Contributions

All authors listed have made a substantial, direct, and intellectual contribution to the work and approved it for publication.

## Funding

This work was supported by grants from the Startup Fund for scientific research, Fujian Medical University (No. 2017XQ1074).

## Conflict of Interest

TLiu is employed by the company Novartis. The remaining authors declare that the research was conducted in the absence of any commercial or financial relationships that could be construed as a potential conflict of interest.

## Publisher's Note

All claims expressed in this article are solely those of the authors and do not necessarily represent those of their affiliated organizations, or those of the publisher, the editors and the reviewers. Any product that may be evaluated in this article, or claim that may be made by its manufacturer, is not guaranteed or endorsed by the publisher.
