# Supplementary figures and images for: Case Report: Successful Treatment of Acute Generalized Exanthematous Pustulosis With Secukinumab
Source: Front Med (Lausanne). 2021 Dec 16;8:758354. doi: 10.3389/fmed.2021.758354 (PMC8725728; doi:10.3389/fmed.2021.758354)

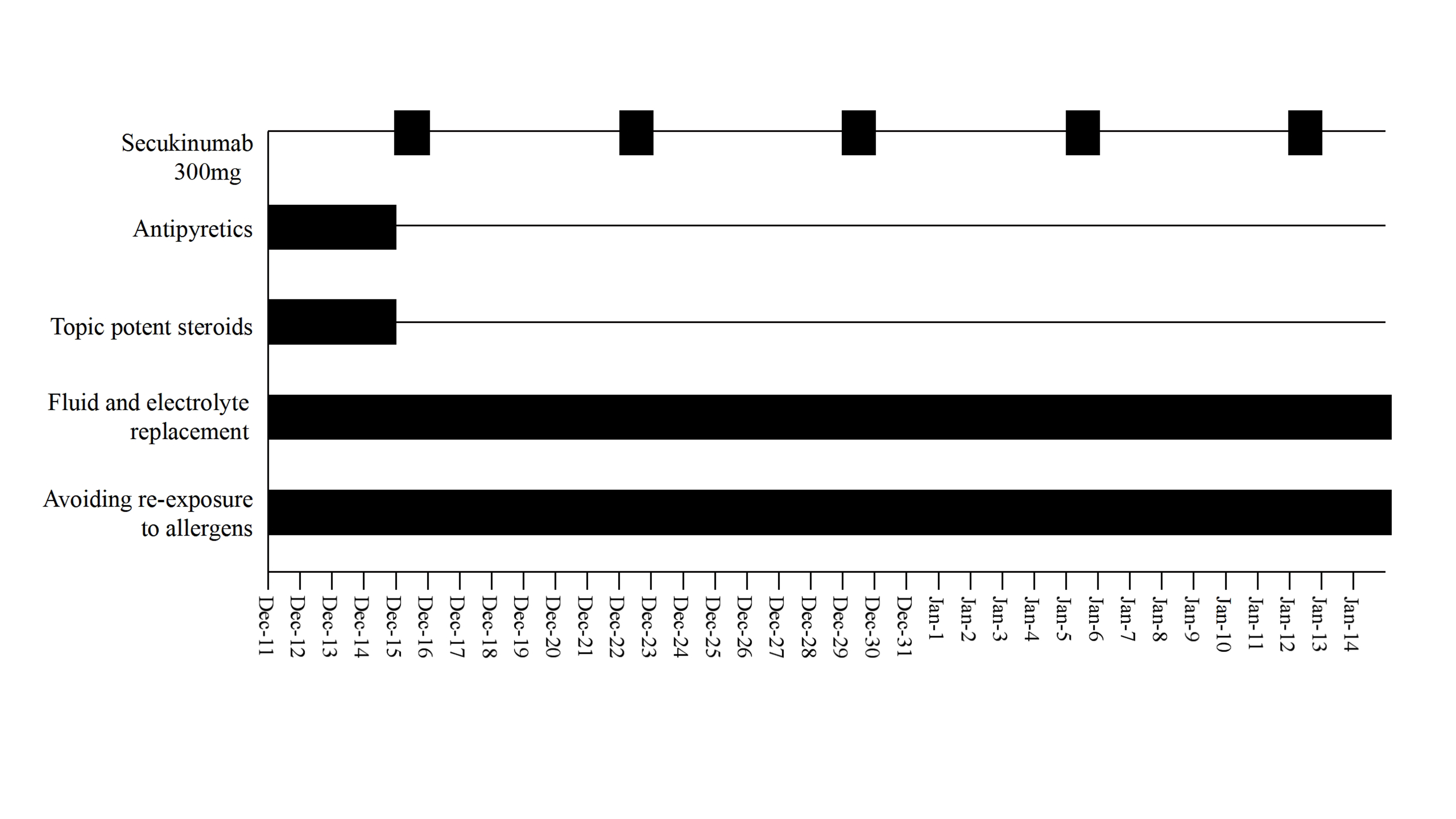

Supplement: Supplementary file 1 [file Image_1.jpg]
